# Discordant indigenous and provider frames explain challenges in improving access to arthritis care: a qualitative study using constructivist grounded theory

**DOI:** 10.1186/1475-9276-13-46

**Published:** 2014-06-11

**Authors:** Wilfreda E Thurston, Stephanie Coupal, C Allyson Jones, Lynden FJ Crowshoe, Deborah A Marshall, Joanne Homik, Cheryl Barnabe

**Affiliations:** 1 Department of Community Health Sciences and Department of Ecosystem and Public Health, University of Calgary, 3280 Hospital Drive NW, Calgary T2N 4Z6, Canada; 2 Department of Community Health Sciences, University of Calgary, 3280 Hospital Drive NW, Calgary T2N 4Z6, Canada; 3 Department of Physical Therapy, Faculty of Rehabilitation Medicine, University of Alberta, 2-50 Corbett Hall, Edmonton T6G 2G4, Canada; 4 Department of Family Medicine, University of Calgary, 3330 Hospital Drive NW, Calgary T2N 4 N1, Canada; 5 Department of Medicine, University of Alberta, 562 Heritage Medical Research Building, Edmonton T6G 2S2, Canada; 6 Department of Medicine and Department of Community Health Sciences, University of Calgary, 3330 Hospital Drive NW, Calgary T2N 4 N1, Canada

## Abstract

**Introduction:**

Access to health services is a determinant of population health and is known to be reduced for a variety of specialist services for Indigenous populations in Canada. With arthritis being the most common chronic condition experienced by Indigenous populations and causing high levels of disability, it is critical to resolve access disparities through an understanding of barriers and facilitators to care. The objective of this study was to inform future health services reform by investigating health care access from the perspective of Aboriginal people with arthritis and health professionals.

**Methods:**

Using constructivist grounded theory methodology we investigated Indigenous peoples’ experiences in accessing arthritis care through the reports of 16 patients and 15 healthcare providers in Alberta, Canada. Semi-structured interviews were conducted between July 2012 and February 2013 and transcribed verbatim. The patient and provider data were first analyzed separately by two team members then brought together to form a framework. The framework was refined through further analysis following the multidisciplinary research team's discussions. Once the framework was developed, reports on the patient and provider data were shared with each participant group independently and participants were interviewed to assess validity of the summary.

**Results:**

In the resulting theoretical framework Indigenous participants framed their experience with arthritis as 'toughing it out’ and spoke of racism encountered in the healthcare setting as a deterrent to pursuing care. Healthcare providers were frustrated by high disease severity and missed appointments, and framed Indigenous patients as lacking 'buy-in’. Constraints imposed by complex healthcare systems contributed to tensions between Indigenous peoples and providers.

**Conclusion:**

Low specialist care utilization rates among Indigenous people cannot be attributed to cultural and social preferences. Further, the assumptions made by providers lead to stereotyping and racism and reinforce rejection of healthcare by patients. Examples of 'working around’ the system were revealed and showed potential for improved utilization of specialist services. This framework has significant implications for health policy and indicates that culturally safe services are a priority in addressing chronic disease management.

## Introduction

In Canada, 4.3% of the population reports Indigenous identity representing First Nations, Inuit and Métis ancestry [1]. Arthritis is the most common chronic disease experienced by Indigenous populations in Canada, and population-based studies estimate that the prevalence of many arthritis conditions is at least 1.3-1.6 times more frequent than that of the non-Indigenous population [2] with high rates of disability observed [3] including rates in the 25–44 year age group [4]. A significant rise in the prevalence of arthritis conditions in the general population is anticipated over the next 30 years [5] and given that approximately half of the Indigenous population is currently under the age of 25 years [1], there will be a great increase in need for arthritis care. It is critical for the musculoskeletal healthcare provider community and healthcare administration to address future capacity issues now, and strategize on how they will increase access to and provide adequate care for an increasing number of Indigenous peoples with arthritis.

Limited work has been done to map current patterns of healthcare utilization for arthritis by Indigenous populations. In one of the few studies, Métis people in Manitoba, Canada were shown to have higher rates of physician visits, hospitalizations and surgeries for osteoarthritis or musculoskeletal disease compared to the general population [6]. Analysis of provincial administrative data in Alberta, Canada, however, revealed that despite a two-fold increase in the prevalence of osteoarthritis, and a two-fold higher use of primary care services for the condition, First Nations people had reduced utilization of orthopedic consultations (standardized rate ratio 0.39, 95% CI 0.38-0.40, p < 0.001) and hip or knee arthroplasty (standardized rate ratio 0.30, 95%CI 0.27-0.33) compared to the general population [7]. The reasons for these patterns remain unexplored to date.

Potential reasons for disparate healthcare utilization for Indigenous peoples have been proposed, although not specifically for arthritis. Because health services are the responsibility of provinces but Indigenous peoples living on reserve are the responsibility of the federal government [8] the focus is often on patient location. The need to travel for services and the lack of provision of more specialized care in rural locations have been identified as concerns [9]. Indigenous peoples in the Northwest Territories, for instance, demonstrated higher use of nursing and social services compared to physician services, reflecting delivery of health services by non-physicians in remote and isolated locations [10]. Data based on a wider population from the 1991 Aboriginal Peoples Survey also revealed that Indigenous peoples, particularly those living on-reserve, were less likely to use physician services compared to the general Canadian population [11]. This may drive low use of specialist care, as physician-to-physician referrals are usually required.

Disparities in healthcare utilization thus underscore the complexities of equity [12]. Racher and Vollman [13] demonstrate that definitions of access to healthcare vary and include multiple dimensions, including potential and realized access; equitable and inequitable; effective and efficient; initiated and continuous; and spatial and aspatial. Thus several issues require consideration when studying access to health services for Indigenous peoples, such as, supply and use; use over time; the fit between consumer and service; the geography of service; subjective and objective data; user and non-user perspectives; definitions of need; the role of outcomes of care; and the interaction of these many factors which creates feedback loops. Kleinman’s seminal model of the health system provided insight into these loops and a way to “make sense of the social and cultural context of healthcare” [14]. He drew our attention to how external social, political and economic factors influence health and the internal structure of local health care systems. Kleinman divided the health care system into 3 sectors: popular, professional and folk. He defined the popular sector as including lay persons, non-professional and non-specialists, constituting the popular culture arena in which illness is first defined and health care activities initiated. The professional sector was characterized as organized healing professions, and includes the medical systems that provide hegemonic evaluative criteria for what makes up a good system. The folk sector is where therapies that are non-allopathic, that is, alternative and complementary, are delivered. Thus, while documenting the differences in rates of healthcare utilization is important for identifying where gaps exist, understanding how to address those gaps requires a more in-depth knowledge of the processes occurring at individual and system levels.

The purpose of this qualitative study was to develop a theoretical framework that would advance understanding the processes, barriers and facilitators to arthritis care, at individual and system levels, for Indigenous peoples in Alberta, Canada. In so doing, we aimed to identify what changes are required to improve delivery of arthritis specialist services to the population at highest risk of the disease and its consequences.

## Methods

### Study design

This qualitative study employed a constructivist grounded theory approach. This methodology was appropriate to this study as it sees knowledge as socially constructed with multiple viewpoints acknowledged among research participants as well as researchers [15]. The research team was multidisciplinary and included specialization in rheumatology, physiotherapy, family medicine, Indigenous health, population health promotion, health services research, and epidemiology. Two members were Indigenous. This was our first project as a team so a methodology that allowed our various viewpoints to be incorporated and reflected upon was important [15]. In addition, data was collected and analyzed to make participants “actions, interpretations, and influences” explicit [15] as was necessary to understanding their healthcare utilization. The methodology was also respectful of Indigenous perspectives on research in that it allowed individuals to tell their own stories and to reflect on the conclusions drawn [16].

### Study participants

Following standard grounded theory methodology, data collection (recruitment and interviews) and data analysis occurred iteratively. Based on Kleinman’s model we considered it necessary to collect data from both people with arthritis and arthritis care providers (hereafter referred to as patients and providers respectively). In order to ensure that we broadly considered viewpoints based on location of residence or practice respectively (urban or reserve), system characteristics, and tribal affiliation were included it was necessary to recruit from more than one region in the province and from a reserve site. Initial recruitment of Indigenous patients took place at various centers in Alberta, Canada, including urban academic practice locations in the two major cities of Calgary (the University of Calgary Division of Rheumatology Clinic) and Edmonton (University of Alberta Hospital Rheumatology Clinic and the Alberta Hip and Knee Clinic), an urban primary care clinic for Indigenous patients (the Elbow River Healing Lodge) and a rural reserve health centre (Siksika Health and Wellness Centre). Calgary and Edmonton are about 300 kilometers apart; Siksika is about 129 km east of Calgary. Indigenous peoples with arthritis were approached by clinic staff for permission to have researchers contact them about the study. Recruitment was also encouraged with posters at the clinics. Eligible participants were ≥18 years of age, and self-identified their Indigenous status and arthritis diagnosis. Healthcare providers were recruited through the clinical members of the research teams’ peer networks. These providers were from a variety of disciplines, including physiotherapy, nursing, primary care physicians, and specialty care physicians (rheumatologists and orthopaedic surgeons). All participants’ identities were anonymous in that the research coordinator did not report to the team the content of their study interview.

Theoretical sampling techniques guided recruitment. We wanted to include patients with a variety of conditions and lengths of illness, and who had experienced different aspects of the continuum of care possible for arthritis patients so that we could explore if there were barriers at different stages. During the analysis additional participants with specific characteristics were sought as the theory emerged and different perspectives were hypothesized [15]. For instance, it was thought that there might be important differences between patients actively engaged in care as compared to those who were not. Recruitment was then expanded outside healthcare facilities to 6 community organizations in Edmonton that did not directly deliver healthcare services. After preliminary data analysis of the healthcare provider interviews, gaps in key arthritis care service areas were identified and further family and specialist physicians were recruited at their respective clinics. Preliminary analysis also showed that many healthcare providers in Calgary and Edmonton in fact had limited experience serving Indigenous peoples. It was hypothesized that providers with more extensive experience with this population may hold different perspectives, and these participants were sought at a family practice, and a physiotherapy clinic in communities other than Calgary, Edmonton and Siksika. Data collection, analysis and recruitment continued in this way until saturation was reached in the analysis; that is, no new information was being identified [17-19].

### Interviews and data analysis

Individual, face-to-face, semi-structured interviews were based on interview guides created for each participant group. The interview guide for patients was focused on the participants’ story about their disease, causes, progression, and their history of seeking health services. The interview guide for providers investigated their experiences in serving Indigenous people with arthritis, their ideas about gaps in health service utilization, as well as their thoughts on the barriers and facilitators to healthcare access for Indigenous peoples.

Interviews were conducted by three research assistants and one of the authors [SC] who received training in qualitative interview methodology [20]. The interviewers explained the study purpose and design to participants during the informed consent process, in person, immediately proceeding the interview. Interviews took place between July 2012 and February 2013 at the clinics or a location of the participants’ choosing. Each interview was recorded and transcribed verbatim; a 10% sample was randomly selected and the audiotape and transcribed versions compared as a quality insurance measure of transcription accuracy. Interview transcripts were uploaded to NVivo 9© for analysis and a separate project file was created for the patients and providers. Field notes, written during and after interviews, were included in the project files. Coding of the data followed standard procedures for grounded theory (i.e., open coding, axial coding to cluster codes into categories, and selective coding to develop themes and concepts) [15]. Initial analysis was performed in parallel by separate researchers [WT & SC] to allow for contrast and comparison. After a draft model was developed the entire research team reviewed the description and debated the interpretations [21]. This was done in videoconferences, teleconferences, in face-to-face meetings and through email with attendance varying. When needed, WT and SC would return to the data to respond to questions and concerns. Several iterations of the model were produced before the results were presented back to the participants. The final model was also provided to the directors and managers of those clinical recruitment sites for which existing long term research agreements with study team members were in place.

### Rigor

Several aspects of the methodology ensured rigor in the study. Analysis was triangulated by having two researchers work separately and then together. This was augmented by having a multidisciplinary team work on analysis and writing with access only to anonymous data. Differences in interpretation were debated and consensus achieved. Member checking was used to validate the analysis. The patient and provider contributions to the model were compiled in separate summary reports and returned to the participants in that group. This was done so as not to engage each group in debating the validity of the other group’s perspectives when they had not seen the data. Rather we wanted to know if the representation of each group’s collective response was seen by the members as accurate. Patients were sent the reports using mail or email based on their preference. Providers were emailed the report. Attempts were made to contact all participants to ask whether the report aligned with their views, if they disagreed with anything, and if there was anything that needed to be emphasized or clarified. Participant feedback was collected by one author [SC] during phone conversations or by email. The feedback was read and discussed by two researchers [SC & WT]. Feedback on the preliminary report was received from 5 patients and 5 providers. All responded positively to the contents relevant to them and did not suggest changes or re-emphasis except in one case. One patient said they would not use the phrase 'toughing it out’ but then later in the phone call discussed using this process. Since others had used this phrase we decided to keep it. Multiple providers asked to see the patients’ view but this was not provided until the study was completed.

Literature, including Kleinman [14], was consulted during the analysis to aid in interpretation [15] and strengthen the reliability and validity of the study. To ensure transparency, quotes from the research participants were used to illustrate key components of the model and demonstrate how perspectives of participants were included in analysis and interpretation.

### Ethics

Ethical approval was granted by the Health Research Ethics Board at the University of Alberta (Pro00022623) and the Conjoint Health Research Ethics Board at the University of Calgary (E-24575). The two researchers who accessed the primary data were not clinicians and this ensured that no patient or provider could be identified by the rest of the team. Care was taken not to reveal identities in the quotations selected for reporting of results. A unique identifier for each participant is used for quotations with IP representing Indigenous Patient and HP, Healthcare Provider.

## Results

### Participants

Indigenous Patients (IP): Sixteen self-identified Indigenous people with arthritis participated, the majority of whom (n = 13) were recruited from the specialty clinics. Three participants were recruited through organizations that did not provide healthcare directly, and 2 of these were not actively receiving care. Our sample included participants from urban and rural areas of both southern and northern Alberta, including 5 males and 10 females, ranging in age from 30 to 73 years. According to patients they had been living with either osteoarthritis or rheumatoid arthritis for <1 year to >20 years. Interviews were 24 to 97 minutes in duration.

Healthcare Providers (HP): Fifteen healthcare providers were recruited in total, including physiotherapists, occupational therapists, nurses (registered nurse, licensed practical nurse, nurse practitioner and case manager), general medical practitioners, orthopaedic surgeons, and rheumatologists. Providers were at various stages of their career, having practiced for anywhere from 3 to 46 years. Interviews with health providers lasted 34 to 90 minutes.

### The conceptual framework

**Figure 1:**
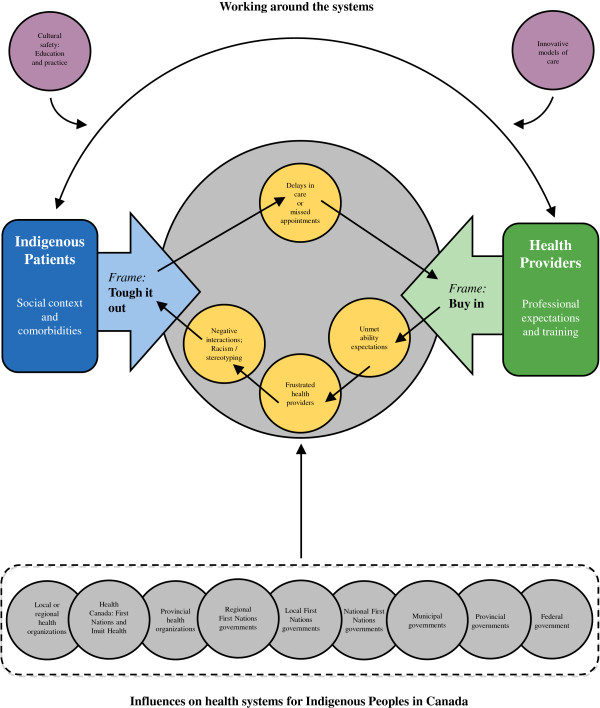
**Health services access: Indigenous Patient and Heath Provider frames.** A theoretical framework which models patient and provider interactions within the healthcare system and illustrates complex contextual factors that influence arthritis care for Indigenous people.

The theoretical framework resulting from the study is depicted graphically in Figure 1. We will begin with an overview and then present the results in more detail.

Consistent with Kleinman [14] the providers and patients described different social contexts that affect how they understand and describe the experience of seeking care for arthritis by Indigenous peoples. For Indigenous patients, interactions directly experienced between themselves and providers, and indirectly experienced between their family members or friends and providers, inform their decisions about accessing various parts of the healthcare system or individual providers. Healthcare providers are largely informed about Indigenous peoples’ experiences with arthritis through their past interactions with patients. The complexity in the provision and governance of healthcare for Indigenous peoples in Canada creates another barrier to care. When providers are able to work around existing systems and structures and create innovative access models that embrace culturally safe environments, utilization can be improved substantially.

#### ***The indigenous patients’ frame: 'tough it out’***

When asked about their disease, patients gave vivid descriptions of the symptoms of arthritis, predominately pain, stiffness and reduction in physical mobility. The following quote from a patient in the early stages of rheumatoid arthritis illustrates the all-encompassing impact of the disease:

“I needed help to get up from bed......I need help to go to the washroom. I need help to bathe myself, like I was literally falling apart I thought I was eh....and, and it was all happening just (snapping fingers) so fast. (30IP)”

The emotional impact of the symptoms and lifestyle changes that impacted them and their family were also evident in the majority of the interviews. The patients described frustration, anger, and depression as a result of their experiences and often, associated with onset of disease.

When the patients discussed their arthritis symptoms and complications of the disease the phrase “toughing it out” was often used. Going to the doctor as soon as one felt pain in one’s joints would not be expected, and delays in seeking medical care were often attributed to this 'toughing it out’ frame. Patients reported that they continued to use 'toughing it out’ as a coping mechanism through the course of the disease. Living with arthritis was predominately described as hard but there was a common story of perseverance, for instance, “the guy [father] showed up what, what strong meant, you know, you gotta, you gotta be strong. You gotta, you never give up” (33IP). One patient also articulated 'toughing it out’ as a traditional teaching that guided life generally. This illustrates that the social context in which the Indigenous patients make their decisions is informed by present day as well as historic factors:

“You know and Kokum used to tell us this story. Indians said in life it’s like going through the trees, the bushes and that and you stumble and you trip over these logs that have fallen and you get scratched and you break this and that and then all of a sudden you come to a clearing, but that’s life, you’re going through this forest and you know this happens, that happens. It’s just, it’s just part of life. It is, you know. And then you come to a clearing, not that that’s the end of it but at least you got through that hardship. That’s just it, everybody has to go through it. Like it or not, you have to go through it. (32IP)”

As the disease progressed, 'toughing it out’ eventually became too difficult. The patients articulated that family members were often influential in the early decision to seek care and in managing their condition. Some people sought relief through traditional medicine and allopathic health care, how this did not seem to play a large role in arthritis care for the majority of participants.

### Social context and comorbidities

Patients in this study were also 'toughing out’ many comorbidities (e.g., diabetes, amputated feet, heart disease, eczema) and not just the pain from arthritis. Additionally, socially and emotionally they were 'toughing out’ painful life events and challenges. The inclusion of side comments about traumatic events was common, revealing families and communities with addictions, mental health issues, physical disability, cancer and other diseases. These were not described as unexpected or unusual events, but were normalized in conversation. Thus, family members may be requiring the patient’s social support at the same time the patient has an appointment or has to make some other decision around treatment of their arthritis. In one case a participant described challenges determining optimal medication for arthritis management while at the same time she battling cancer and was assisting her nephew with stressful financial challenges. Eleven of the patients named a relative that also had arthritis, in fact, naming more than one was common and 3 people named five or more family members. Sometimes these family members were also 'toughing out’ arthritis symptoms. Thus, arthritis was constructed as a disease that was so common that it was normalized, as were other sources of suffering.

#### ***The provider frame: lack of 'buy in’***

All of the professionals were trained and had practiced a minimum of three years. They exemplified the professional sector as described by Kleinman [14]. They were working in complex health care systems that were regularly under evaluation and scrutinized by government and non-government organizations for efficiency and competency. Attention to the needs of Indigenous peoples was covered by a relatively small portfolio in the provincial health system called the Aboriginal Health Program that had only recently begun education in cultural safety. Thus, the practice frame was based on expectations developed primarily for non-Indigenous patients, and professional expectations from professional bodies and governance structures.

Most providers who saw a large number of Indigenous patients described an increased severity of arthritis in them compared to non-Indigenous patients: “I find that they often present with more severe disease, ah, more advanced disease, untreated disease” (08HP). From the provider point of view, patients lacked 'buy in’ and they often stated that if the patients had more knowledge of arthritis and potential therapies their 'buy in’ would increase. Providers believed that Indigenous peoples didn’t understand the value of health services, or believe that treatments would improve their quality of life, and didn’t trust the recommendations of providers:

“I think it might have to do with access [to health services] but even if the access were there, it, it might also have to do with their own buy in. Um, like do they, do they feel that the health professionals that are, that they do see are actually going to be helping them, will they seek the help? (02HP)”

Providers reflected on the impact of delayed presentation of illness on their role as care providers. They saw living with the disability of pain and joint dysfunction as unnecessary and unacceptable. The poor condition of patients at presentation was perceived as a preventable result of patients’ actions and inactions. This created frustration for well-intentioned providers that would become a source of tension between them and Indigenous peoples from the first appointment.

### Professional expectations and training

Most providers thought that much of Indigenous peoples’ lack of 'buy in’ could be resolved by education to ensure Indigenous peoples understand the inherent value of specialists and services in improving their quality of life or alleviating their symptoms. The following quote exemplifies the argument that knowledge will lead to what are viewed as rational decisions:

“I think there’s a huge educational component to it and I don’t mean teaching physicians how to spot arthritis. I think teaching Aboriginal individuals that there is better treatment available and using it is, is not a submission to anything other than smart behavior. (43HP)”

Except for those professionals working in an Indigenous health centre, participants placed less emphasis on the historic, social, and cultural factors that differentiate Indigenous patients from non-Indigenous patients.

#### ***Health systems: experiences with care***

The interactions between patients and providers that informed their framing of Indigenous experience with access to arthritis care took place within health systems. Past experiences with providers were mentioned by more than a third of patients, and patients recruited from the community were particularly vocal about the racism they had experienced in those encounters. Once patients sought care, their arthritis stories evolved as an interaction between their experience of symptoms and treatment received (both medical treatment and behaviour of providers). Once in care their assessments of their arthritis and their views of healthcare depended on the responses they received from providers. Some patients discussed positive relationships with family physicians who had facilitated referral to arthritis specialists and ironically, given the more advanced state of their disease and lack of cultural safety in the system, most of the participants were satisfied with the care they were currently receiving. This could rapidly change, however, as one participant explained that having seen a rheumatologist twice she missed appointments because of her job and that doctor then refused to see her again. She waited until moving to another city to get a new referral to another rheumatologist. Another patient described the impact of a negative interaction with a provider who offended them:

“I find the best thing for me to do is just walk away because Lord knows I don’t, I don’t take kindly to people that treat me that way or anybody else for that matter and the best thing was just to walk off. (32IP)”

Among providers, the most salient evidence of lack of 'buy in’ experienced through missed appointments, a topic that was commonly raised. Not surprisingly, appointments were much more salient issues for providers than for patients, as appointments are also a means of ordering provider work. Appointments were discussed by providers along the pathway of arthritis services (primary care, allied health and specialists) who shared perceptions that Indigenous peoples more frequently miss appointments:

“I guess the fact is for a certain segment of our population, you cannot assume that they’re going to come back, you know, so if you have something that you need to tell them, you know, it’s very problematic. (29HP)”

Providers discussed the perceived consequences of missed appointments. Often viewed as a missed opportunity for health provision, a loss of continuity of care, or a miscommunication between patients and providers, missed appointments caused frustrations for providers that became linked to stereotypes. The consequence may be a subtle change in how Indigenous peoples are viewed as articulated by a participant from a primary care service:

“Lots of people don’t understand it so our patients are a no show, the non-compliant word comes out and, ah, and they won’t rebook them so we have to try to rebook them somewhere else, like there’s no flexibility within the system. That’s the big thing. I guess is another big thing is there’s no flexibility. (28HP)”

This is reinforced by the response from one provider when asked about the influence of health attitudes on arthritis care: “Yes I think because of historically there have been so many challenges, that, that you start to develop an impression and, and that’s a barrier” (02HP) to arthritis care provision.

### Influences on health systems for indigenous people in Canada

External political factors influence health and the internal structure of local health care systems [14]. Although participants did not describe this concept explicitly, the research team recognized in the stories and in the provider interviews that these systems contribute to complexities in arthritis care provision. It is beyond the scope of this paper to describe the funding structures and policy mechanisms that are in play, however, at the patient and system level they result in confusion over how services will be paid for. Provincial governments provide universal insured health services to all citizens to cover hospital and some outpatient services. First Nations living on-reserve and Inuit are provided some medical supplies, some prescription drugs and medical transportation as specified by the Non-Insured Health Benefits program. First Nations patients living off-reserve and Metis populations can only acquire these benefits by purchasing them independently or through their employer or school [8]. The actual provision of services on-reserves is variable, as is the actual provision of uninsured benefits and Indigenous people who are unregistered, Métis, or living in a city have an even more complex environment [9].

Although many providers did recognize geographic, financial or social barriers to accessing care, they realized that the structure and expectations of the health systems did not allow them to take these into account in providing Indigenous peoples care:

“And sometimes I think, you know, is it time, is it, you know, because we schedule 15 minute or 30 minute appointments? Is it not enough time to explain or listen to the story correctly? (41HP)”

Two clinics that focused uniquely on Indigenous care described taking the opportunity to work around the systems to try new ways to improve access. The clinic in Siksika, for instance, made 'drop-in’ or unscheduled appointments available partially to offset the emphasis on appointments, and the Elbow River Healing Lodge had an outreach worker who was available to address the needs of clients in the community.

## Discussion

The strengths of this study include the methodological coherence and attention to rigor. The multidisciplinary team was a real strength in triangulating the analysis. In addition, sampling from more than one site, with two cities, including both patient and provider views, and covering more than one reserve in Alberta helped ensure there is greater transferability of results. Of course, the alternative weakness is that the research took place within one provincial health system and a context where access to health care is universally available and these may not represent other locales. This is the major weakness of the study but we tried to provide as much information as possible, within the limitations of space, for others to assess the applicability of the results to their locales. Many procedures were employed to ensure participant comfort; however, the limited discussion of use of traditional medicines may indicate that they did not feel completely free to discuss openly. On the other hand, many sensitive topics were discussed, so the alterative explanation is that traditional medicines are not widely used. We do not feel that this information was critical to our model; however, a new study by team members will more thoroughly investigate the question of traditional medicine use.

This study makes an important contribution to the scant literature examining arthritis healthcare utilization for Indigenous peoples. The results can also be useful in understanding access for other chronic diseases requiring involvement of specialists. Arthritis is a chronic disease that can lessen quality of life directly through the pain and disability experienced, and indirectly through limitations on the ability to work and to enjoy other activities. While the biomedical disease may follow similar paths in Indigenous and non-Indigenous peoples of a similar age and background, in the Indigenous population the imbalance in social determinants of health are factors that create additional complexity in management. The tendency to place responsibility on patient attitudes is not helpful when it is systemic factors that create barriers to relationship development among patients and health care providers.

The results help explain low utilization rates for specialist care among Indigenous people within the context of a continuum of care. It appears this is not driven by cultural and social preferences for non-specialist care, but rather by prior negative experiences with racism in the healthcare system. Others have shown that health sector discourses and practices around evidence-based practice in medicine, have contributed to colonization and marginalization [22]. Methodological biases in research preclude evidence based on “tradition, convention, belief, or anecdotal evidence” [22]. In addition, based on western traditions of science and evidence, evidence-based practices have rarely been tested with Indigenous populations, yet when they don’t respond like non-Indigenous people, they are viewed as having deficits. A general criticism of health system reform from a health promotion perspective is that risk factor epidemiology continues to be the dominant paradigm in North America, with a focus on changing individual behaviors rather than addressing the social and structural determinants of health [23].

We have shown that models of care that assure innovation around colonial systems and cultural safety are valued by both patients and providers and provide a means to achieve equitable health outcomes. In fact, the reports of some participants suggested that policies around creating culturally safe relationships and environments in health care settings may be the priority for simultaneously engaging and retaining patients in care.

Families emerged as an important factor in utilization of arthritis care. The Indigenous peoples in this study revealed families and communities with many other health conditions, and as found by others Indigenous people may prioritize family, friends and community needs over their own health [24]. Participants described some circumstances (e.g., a funeral in the community) in which the Indigenous patients chose to attend to those social obligations over an appointment with a healthcare provider. It is also important to note that the cultural values of putting family and community first are among those that have kept Indigenous peoples resilient in the face of repression, oppression and repeated attempts to assimilate them. This highlights that arthritis care strategies must incorporate a broader view of the 'patient’ to include the familial support systems.

In Kleinman’s model [14] the professional sector has a strong influence on how health and health care are understood and valued and this was illustrated by the assumptions about accessing allopathic health care that were implicit in the study and deserve the label hegemonic. The first assumption was that allopathic health was acceptable and desired. The second was that appointments with specialists were valued resources. The healthcare provider frame was centered on the idea that the 'buy in’ of Indigenous peoples’ had to be fixed. The providers assumed that Indigenous peoples had knowledge deficits (not knowing enough about arthritis as a disease or of the effectiveness of certain treatments), cultural deficits (not appreciating the value of an appointment), and resource deficits (transportation), among others. This frame borrows from the deficit model, placing the responsibility on individual limitations, and assuming weaknesses in individuals or communities [25]. This bias towards individual patient level rather than systemic solutions was reported in another study where providers who were asked about barriers to renal transplantation focused on language issues and cultural factors [26]. As reported, “this propensity to locate the problems with the patients rather than in the interaction with the system or the system itself might de-emphasize modifiable factors that may be hindering Aboriginal patients from engaging in their treatment” [26]. It is notable that the healthcare providers did not query what the deficits were in their various health professions (family physician, surgeon, physiotherapist, pharmacist, and so on) that may account for their failure to *attract* Indigenous peoples to their practice.

Examination of the provider frame based on hegemonic assumptions points to underlying ability expectations held by health providers. Wolbring discusses how ideas, described by the deficit model, can form ability expectations that become normative and slide into ableism, which is associated with prejudice and discrimination [27]. For example, when wanting people to keep an appointment morphs into viewing this as an essential ability, the result is ableism. This ability expectation lens aids in understanding how inequities can be reinforced within the healthcare system and the building up of stereotypes of Indigenous patients as disrespectful, unreliable, and so on. It is easy then to generalize these characteristics to all Indigenous peoples, thus reinforcing racism. This is made more possible in a provider culture where failed appointments are seen as a drain on scarce resources [28]. A negative impact of non-attendance by patients on the patient-provider relationship has been demonstrated in other contexts [29]. The type of personally-mediated racism described in the study is often unconscious and unintentional [30]. Healthcare settings provide conditions for stereotyping of minority members even by well-intentioned health providers [31]. Stereotyping, bias and uncertainty have been found to contribute to health disparities for other minority populations, and were also linked to healthcare systems and the legal and regulatory processes surrounding health services [32]. The deficit model is actually detrimental to Indigenous peoples because it can reinforce existing apathy and neglect by providers [25]. Thus, utilization is better explained by biases, stereotyping and discrimination experienced. Therefore, achieving equity in arthritis care will depend on the broader availability of culturally safe systems, rather than changes in individual Indigenous peoples or providers.

Indigenous people in New Zealand face similar patterns of health disparities in both health status and access which have been linked to privilege and deprivation [33]. Improving access to arthritis services is not just a task for health systems, but calls for other systems (e.g., education, legal, social welfare) to remove differences in privilege and deprivation. As health promoters have found, this call for interdisciplinary work across sectors is common, but little success has been achieved in the efforts [34]. Nevertheless, the Ottawa Charter for Health Promotion [35] remains the best framework for facing these dilemmas with recommendations for building healthy public policy; creating supportive environments; strengthening community action; developing personal skills; and reorienting health services.

## Conclusion

This qualitative study improves understanding of arthritis healthcare use among Indigenous peoples, and adds to scant literature in this field. Analysis of qualitative interviews showed how hegemonic assumptions around healthcare can lead to stereotyping. The resulting framework reveals how low specialist care use by Indigenous patients may be driven by prior experiences of racism. Although 'toughing it out’ may be an important survival skill for marginalized and oppressed peoples, providing arthritis services that incorporate the family in a patient care plan and ensure cultural safety may facilitate the care pathway for Indigenous patients. Health systems must be re-oriented to keep the patients as the centre of focus of care, in order to achieve their aim of optimal health outcomes. Addressing arthritis care reform will necessarily require improvements in social determinants of health for Indigenous population.

## Abbreviations

IP: Indigenous patient; HP: Health provider.

## Competing interests

The authors declare they have no competing interests.

## Authors’ contributions

WT provided expertise in Constructivist Grounded Theory methodology, contributed to the study design, wrote the interview guide, supervised data collection, conducted preliminary data analysis, facilitated team data analysis, and drafted the manuscript. SC carried out a portion of the interviews, coordinated data collection, participated in data analysis, and assisted with drafting the manuscript. CB and AJ assisted with study design, recruitment of participants and supported data collection. All authors assisted in interpretation of the data during team analysis, reviewed drafts for content and read and approved the final manuscript.

## Authors’ information

Wilfreda Thurston is a social epidemiologist who focuses on the experiences of marginalization and discrimination in population health promotion. She has extensive research and practice experience with the Aboriginal population, including homelessness studies, prenatal depression, structural violence and youth. Stephanie Coupal is a research coordinator interested in health services research. Allyson Jones is a physiotherapist and researcher who is interested in preventing the progression of conditions such as arthritis. She is an Alberta Innovates Health Solutions Population Health Investigator. Lynden Crowshoe is an academic family physician with a clinical and research focus on Aboriginal populations, chronic disease, health models and medical education. He also works at the Elbow River Healing Lodge, a special urban health service for Aboriginal peoples. Deborah Marshall is a health services researcher with particular interest in methods for measuring patient preferences in the evaluation of medical interventions and health system evaluation using operations research methods such as system dynamics modelling in musculoskeletal disease. Deborah Marshall is Canada Research Chair, Health Systems and Services Research and Arthur J.E. Child Chair in Rheumatology Outcomes Research. Joanne Homik is a rheumatologist practicing in Edmonton, Alberta. Cheryl Barnabe is a rheumatologist who has opened special clinics on a First Nation in Southern Alberta and at the Elbow River Healing Lodge in Calgary. She is Chair of the Group for Research with Aboriginal Peoples for Health (GRAPH), Institute of Public Health, and holds the Canadian Rheumatology Association - The Arthritis Society Clinician Investigator Award.
